# *Ab initio* insights into hydrogen-rich polynuclear non-metallic superalkali cations

**DOI:** 10.3389/fchem.2026.1827143

**Published:** 2026-06-05

**Authors:** Ambrish Kumar Srivastava

**Affiliations:** Department of Physics, Deen Dayal Upadhyaya Gorakhpur University, Gorakhpur, Uttar Pradesh, India

**Keywords:** Ab initio methods, cations, hydrogen, ionisation energy, superalkali

## Abstract

Superalkalis are defined as the species having lower ionisation energies (IEs) than alkali atoms or the cations having lower electron affinities than alkali cations. Typical examples include FLi_2_^+^, OLi_3_^+^, NLi_4_^+^, etc., which can be represented by the general formula of XM_*k*+1_, where X is a central electronegative atom of valency *k*, and M represents alkali metal ligands. Enormous progress has been made in the research and development of novel superalkalis due to their various applications, including as strong reducers. However, most of the superalkali cations (except a few) reported, thus far, are decorated with alkali metal atoms. Later, binuclear non-metallic superalkali cations were reported in which the role of alkali atoms was played by hydrogen atoms. This led to an inquiry into whether it is possible to reduce the, IE of H-based superalkali species further by increasing the chain, i.e., going from binuclear to polynuclear species. We have performed a series of works in this direction using state-of-the-art computational methods. In addition, hydrogen-based organic superalkalis have also been reported. The present account focuses on some interesting findings of these works and their implication for future directions.

## 1 Introduction

Among all the elements in the periodic table, alkali metals form an ideal group following well the trends in properties, starting from lithium (Li) to caesium (Cs). Common properties of the group include the *n*s^1^ valence configuration, large atomic radii, high chemical reactivity, etc. Their story begins with the loss of their lone valence electron to form the +1 oxidation state, due to having the lowest ionisation energy (IE) among all the elements known to date. Their IEs range from 5.39 to 3.89 eV for Li to Cs, respectively (Lide, 2004). Due to collective effects, however, the IE of some species can be reduced below the threshold for alkali atoms. In 1982, Gutsev and Boldyrev made the first attempt to demonstrate theoretically that certain species may possess a lower IE than alkali atoms and named them ‘superalkalis’ (Gutsev and Boldyrev, 1982). They proposed that certain radicals such as FLi_2_, OLi_3_, NLi_4_, etc., become superalkalis, which can be represented by the general formula of XM_*k*+1_, where X is a central electronegative atom of valency *k*, and M represents alkali metal ligands. According to octet rule (Langmuir, 1919), molecular species tend to achieve noble gas configurations to become stable. For instance, FLi_2_, with 11 valence electrons, give up one electron easily for stability, having a low IE (3.78–3.91eV) and becoming a superalkali cation. Superalkalis act as superatoms (Khanna and Jena, 1995) as they mimic all the properties of alkali atoms with even higher potency. Their strong electron-donating nature makes them powerful reducing agents capable of reducing and activating small molecules such as CO_2_ (Srivastava, 2018a; Srivastava and Srivastava, 2022; Zhao et al., 2017), NO_x_ (Srivastava, 2018b) and N_2_ (Park and Meloni, 2018). In addition, they find numerous applications such as in the design of hydrogen storage materials (Pan et al., 2012; Srivastava, 2024; Wang et al., 2014), superbases (Srivastava and Misra, 2015a; Srivastava and Misra, 2015b; Srivastava and Misra, 2016a) and supersalts (Giri et al., 2014; Li et al., 2008; Srivastava and Misra, 2016b; Yang et al., 2012). Ever-increasing scope of superalkalis makes their exploration contemporary to date. An account of all these aspects has been well documented in the literature (Sun and Wu, 2019).

**FIGURE 1:**
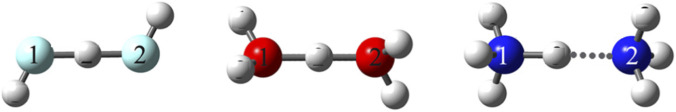
Equilibrium structures of X_2_H_2*k*__+1_^+^ superalkali cations taken from Ref (Hou et al., 2013). where X = F (cyan), O (red), N (blue) and H (grey).

After typical mononuclear superalkali species (Rehm et al., 1992; Zhao et al., 2017), the superalkali cations with more complex structures, including binuclear (Sun et al., 2019; Tong et al., 2011), polynuclear (Liu et al., 2014; Tong et al., 2013), organic (Giri et al., 2016; Reddy and Giri, 2016), and aromatic (Sun et al., 2013) architectures, were explored. However, it was reported (Tong et al., 2011) that the IE of binuclear X_2_Li_2*k*+1_ superalkalis is lower than that of corresponding mononuclear XM_*k*+1_ superalkalis. Note that most of the superalkalis (except a few) reported are decorated with alkali metal atoms. Hou et al. (Hou et al., 2013) replaced the lithium atom in X_2_Li_2*k*__+1_^+^ superalkali cations by a hydrogen atom and reported binuclear non-metallic X_2_H_2*k*__+1_^+^ superalkali cations as shown in Figure 1. This can be expected due to the fact that the H-atom also belong to the alkali metal group in the periodic table, based on its valence electron. The IE of H-based superalkali species is lower than that of parent mononuclear species. This led to an important question: whether it is possible to reduce the IE of H-based superalkali species further by increasing the chain, i.e., going from binuclear to polynuclear species. This article focuses on the works performed in this quest and their implication for future directions.

## 2 Computational note

To assess the superalkali properties of species, the calculation of IE is crucial. The IE can be calculated by the difference of the total electronic energies of the cations and corresponding neutral species, both in their equilibrium structures, which can be obtained by any suitable computational scheme. In practice, however, the vertical electron affinities (VEAs) are used to predict the superalkali nature, which can be calculated by the difference of the total electronic energies of the cations and corresponding neutral species, both at the equilibrium structure of cations. This is evidently due to the fact that these species have primarily been detected using various mass spectrometric techniques such as laser ablation, electron impact ionisation, thermal and surface ionisation, and Knudsen effusion cell methods in the form of cations (Veličković et al., 2007; Veljković et al., 2012; Yokoy et al., 2000). Most of the results presented in this article, unless specified, are obtained by *ab initio* method using second-order Moller-Plesset perturbation theory (MP2) (Møller and Plesset, 1934) and 6–311++G (d,p) basis set using the Gaussian 09 (Frisch et al., 2009) program. The partial charge analyses have been performed using the natural bond orbital (NBO) scheme (Reed et al., 1985) with NBO 3.1 program (Glendening et al., 1998).

## 3 Hydrogen-rich polynuclear non-metallic superalkali cations

### 3.1 Inorganic species

**FIGURE 2:**
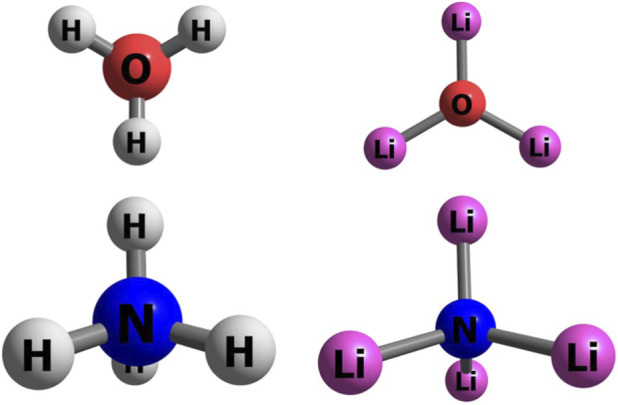
Equilibrium structures of OH_3_^+^, NH_4_^+^, OLi_3_^+^, and NLi_4_^+^ cations taken from Ref. (Srivastava et al., 2020).

According to Gutsev and Boldyrev (Gutsev and Boldyrev, 1982), ammonium (NH_4_^+^), a non-metallic analogue of NLi_4_^+^, should also be considered as a superalkali cation. What about the superalkali nature of FH_2_^+^ and OH_3_^+^? The preliminary calculations did not provide any such evidence. In fact, FH_2_^+^ has sufficiently high VEA (>6 eV). A comparative analysis on OH_3_^+^ and NH_4_^+^ (Srivastava et al., 2020) performed by us using different computational schemes does provide VEA of OH_3_^+^ in the range 5.17–5.25 eV against the reported value of 5.27 eV (Talbi and Saxon, 1989) and of NH_4_^+^ in the range 4.36–4.50 eV against the experimental value of 4.62 eV (Fuke et al., 1994). The structures of OH_3_^+^, NH_4_^+^, OLi_3_^+^, and NLi_4_^+^ are displayed in Figure 2. Although the VEA of OH_3_^+^ also lies just below the border, we do not have much emphasis on this. On the other hand, NH_4_^+^ not only possesses low VEA but also resembles other properties of NLi_4_^+^. For instance, its structure is a tetrahedron (see Figure 2), and it forms a supersalt, similar to NLi_4_^+^. Thus, one can expect, at least in principle, some metallic behaviour in NH_4_^+^, similar to other alkali metal cations. Some reports (Ramsey, 1967; Stevenson, 1975) confirm that metallic NH_4_^+^ indeed exist, but at high pressure. Comparing the VEA of FH_2_^+^, OH_3_^+^ and NH_4_^+^ with those of their binuclear analogues (see Figure 1), one can notice a significant reduction. It seems, therefore, interesting to analyse the trend of VEA of their polynuclear analogues.

#### 3.1.1 F_*n*_H_*n*+1_^+^ series

**FIGURE 3:**
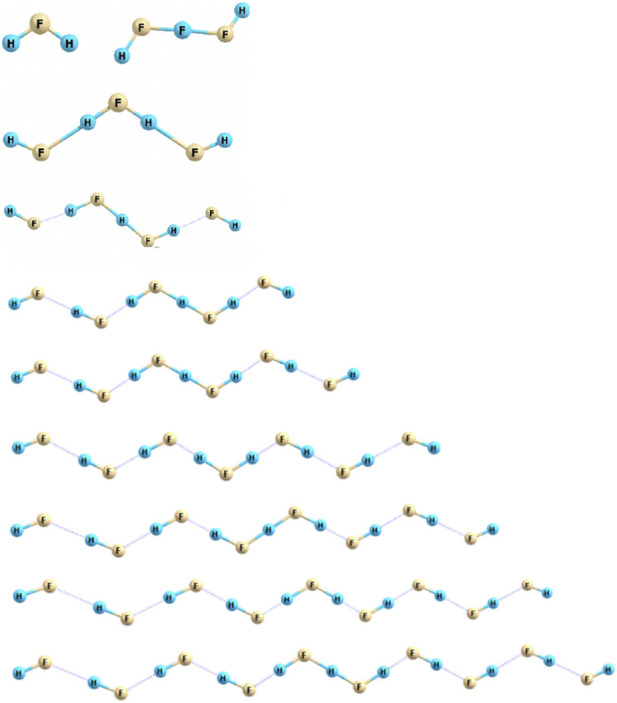
Equilibrium structures of F_*n*_H_*n*+1_^+^ series of cations (*n* = 1–10) taken from Ref. (Srivastava, 2019a).

We first studied the F_*n*_H_*n*+1_^+^ series of cations (Srivastava, 2019a) for *n* = 1–10 using MP2 and *ab initio* molecular dynamics (AIMD) simulation (Iftimie et al., 2005). FH_2_^+^ is a bent structure in which both H-atoms contain a positive charge, and F is negatively charged, unlike some previous assumptions (Hennecke, 2013; Struble et al., 2013). This supported a recent report (Christe et al., 2017), suggesting fluoronium ions with positively charged F as only an artefact. To obtain the structure of F_*n*_H_*n*+1_^+^cations for *n* ≥ 2, the H-atoms in FH_2_^+^ were replaced successively by FH_2_ moieties. The resulting 1D chain-like structures of F_*n*_H_*n*+1_^+^cations are displayed in Figure 3. One can see that these structures appear to possess FH_2_^+^ as the core for odd *n* and F_2_H_3_^+^ as the core for even *n* values, in which additional HF units are attached via H-bonds (F–H···F). Thus, these structures are evolved by adding HF units to these cores.

The energetic stability of F_*n*_H_*n*+1_^+^cations was confirmed by calculating dissociation energy against deprotonation and the HF-reduction. Both dissociation energies are positive, indicating that the cations are strongly bound and difficult to break apart. Further, the deprotonation energies and HF-reduction energies increase with an increase in *n*, leading to an increase in the stability with the length of the chain. The kinetic stability was confirmed through AIMD simulations. The 1 ps trajectory of F_3_H_4_^+^ was analysed at various temperatures from 0 K to 500 K. The snapshots exhibit no structural collapse, and chains remain intact even at high temperatures.

The VEA of FH_2_^+^ is calculated to be 6.08 eV. Evidently, it is not a superalkali unlike FLi_2_^+^. As we increase the length of the chain, VEA starts decreasing such that it becomes 4.48 eV for *n* = 2, consistent with the report of Hou et al. (Hou et al., 2013). Beyond it, the VEA goes on decreasing and becomes as low as 1.60 eV for *n* = 10. This is due to an increase in the positive charge on the FH_2_^+^ or F_2_H_3_^+^ core. Therefore, F_*n*_H_*n*+1_^+^cations belong to the series of superalkali cations for *n* > 1. Since the VEA increases with an increase in *n*, we have analysed the statistical correlation between VEA and *n*. This led to an exponential relation with the correlation coefficient of 0.9958.

$$\text{VEA}=1.5366+6.4583\;\exp\left(‐0.3706n\right)$$

This relation not only provides an upper bound estimate but also predicts an approximate VEA for any larger value of *n*.

#### 3.1.2 O_*n*_H_2*n*+1_^+^ series

**FIGURE 4:**
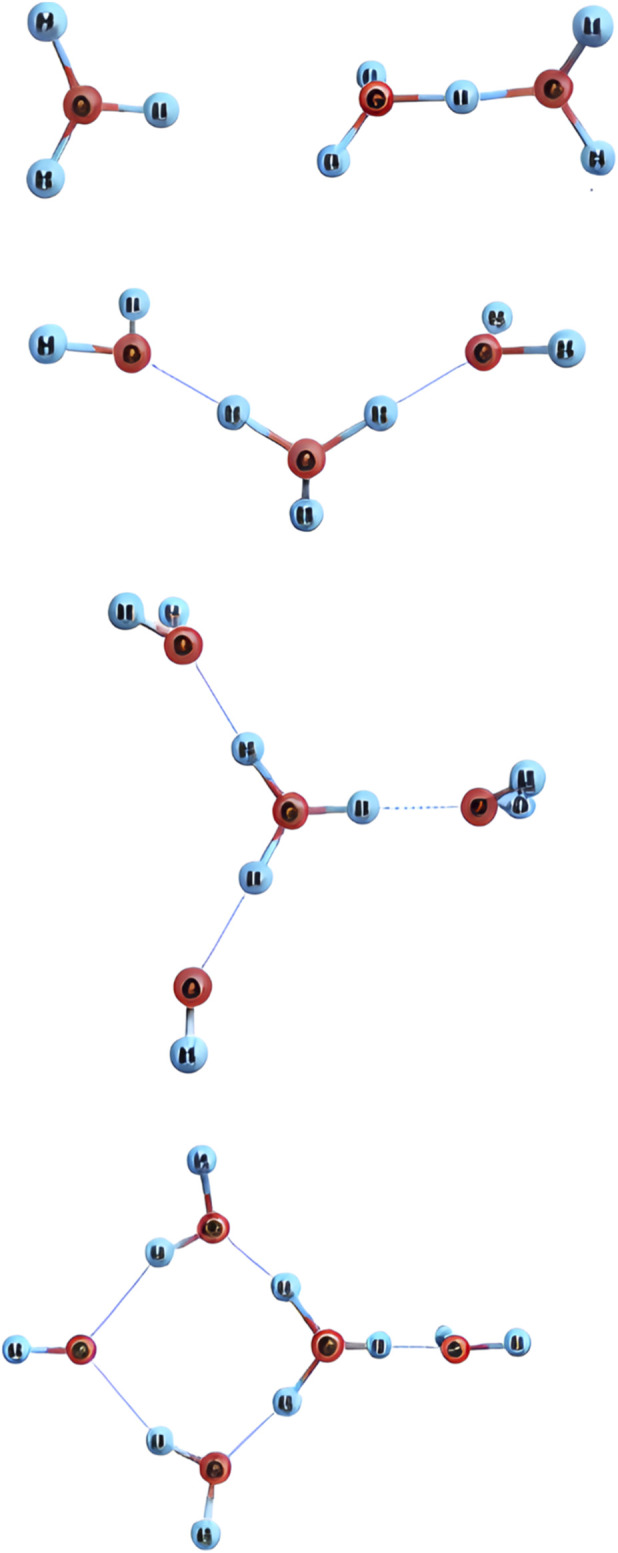
Equilibrium structures of O_*n*_H_2*n*+1_^+^ series of cations (*n* = 1–5) taken from Ref. (Srivastava, 2019b).

Subsequently, we studied the O_*n*_H_2*n*+1_^+^ (*n* = 1–5) series of cations (Srivastava, 2019b) using the MP2 method and quantum theory of atoms in molecule (QTAIM) approach (Bader, 1994; Kieth, 2019). OH_3_^+^ has a trigonal pyramidal Eigen structure, unlike trigonal planar OLi_3_^+^. The successive addition of OH_3_ in the place of H leads to the O_*n*_H_2*n*+1_^+^ (*n* = 1–5) series as shown in Figure 4. O_2_H_5_^+^ forms the classical Zundel cation, where a proton is symmetrically shared between two water molecules as reported in previous studies (Okumura et al., 1986; Okumura et al., 1990; Yeh et al., 1989; Zundel, 1999), including Hou et al. (Hou et al., 2013). For *n* ≥ 3, the O_*n*_H_2*n*+1_^+^energetically favour Eigen-type structures, consisting of a central OH_3_^+^coresurrounded by H_2_O molecules, which are stabilised through multiple hydrogen bonds. However, there exist multiple isomers such as Zundel-type, ring, branched and chain structures for *n* = 4 and 5 (not shown in Figure 4).

The stability of O_*n*_H_2*n*+1_^+^cations was confirmed by calculating their deprotonation and H_2_O-reduction energies, which are all found to be positive and increasing with an increase in *n*. We have also assessed the nature and strength of H-bonds in the O_*n*_H_2*n*+1_^+^series using the QTAIM analysis. Our analysis revealed that these O_*n*_H_2*n*+1_^+^cations are stabilised by partially covalent (medium) and electrostatic (weak) H-bonds. As the number of O atoms increases, the number of partially covalent H-bonds also increases, which leads to greater stability as *n* increases.

The VEA of OH_3_^+^ is calculated to be 5.16 eV, which is lower than that of Li. For O_2_H_5_^+^, this value is reduced to 3.87 eV, consistent with the previous report (Hou et al., 2013). With further increase in *n*, the O_*n*_H_2*n*+1_^+^series, the VEA decreases up to 2.78 eV for *n* = 5. It is obvious that the VEA can be further reduced by increasing the number of *n*, which, in turn, results in an increase in the number of H-bonds due to the presence of more H_2_O moieties. To estimate the limit of VEA, we solvated hydronium into water and calculated the VEA in aqueous medium using the polarizable continuum model (PCM) (Barone et al., 1997; Cossi et al., 1996). This value is found to be 1.85 eV, suggesting the possibility that the series may be extended to reach the value of approximately 1.8 eV, which may not be reduced further.

#### 3.1.3 N_*n*_H_3*n*+1_^+^ series

**FIGURE 5:**
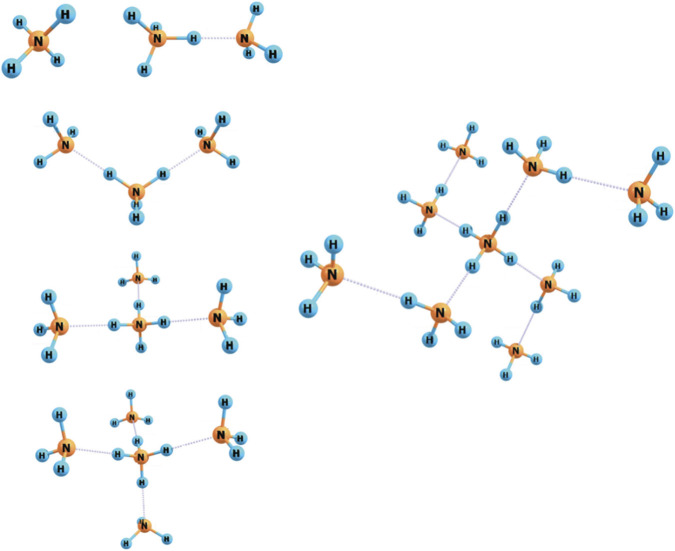
Equilibrium structures of N_*n*_H_3*n*+1_^+^ series of cations (*n* = 1–5) taken from Ref. (Srivastava, 2019c). The structure of N_9_H_28_^+^ is also shown.

As mentioned earlier, NH_4_^+^ can be considered as superalkali cation. However, binuclear N_2_H_7_^+^ have even a lower VEA and therefore, a stronger superalkali as reported by Hou et al. (Hou et al., 2013). In order to form the N_*n*_H_3*n*+1_^+^ series (Srivastava, 2019c), we substituted the H-atom in NH_4_^+^ by NH_4_ moieties successively. The resulting optimised structures of N_*n*_H_3*n*+1_^+^ cations are displayed in Figure 5. One can note that these species can be expressed in the form of [NH_4_···(*n*−1)NH_3_]^+^ complexes, which are stabilised by N–H···N hydrogen bonds. QTAIM analysis revealed that these H-bonds are partially covalent and ionic with medium and weak strengths, respectively. The energetic stability of these cations has been verified against deprotonation and loss of ammonia (NH_3_), and it was found that, like other series mentioned above, the stability of N_*n*_H_3*n*+1_^+^cations increases with an increase in *n*.

Our calculated VEA of NH_4_^+^ is found to be 4.39 eV, consistent with previous results (Gutsev and Boldyrev, 1982; Srivastava et al., 2020). With an increase in *n*, the VEA decreases monotonically from 3.52 eV (*n* = 2) to 2.39 eV (*n* = 2.39), making all of them strong superalkalis. The series can be extended to find larger cations with lower VEA values. For instance, the structure of N_9_H_28_^+^ (see Figure 5), which is stabilised by four partially covalent H-bonds and four weaker electrostatic H-bonds, possesses the VEA of 1.84 eV. The decrease in the VEA due to an increase in NH_4_ moieties can be understood on the basis of charge accumulation of the central atom. It was found that there exists a linear correlation between VEA of N_*n*_H_3*n*+1_^+^and charge on central N-atom (*Q*_c_) with a correlation coefficient of 0.9941.

$$\text{VEA}=14.88+12.89\cdot Q_{c}$$

Like the F_*n*_H_*n*+1_^+^ series, the VEA of the N_*n*_H_3*n*+1_^+^ series also leads to an exponential relation with a correlation coefficient of 0.9976.

$$\text{VEA}=1.70797+3.78896\;\exp\left(‐0.35536n\right)$$

This relation can be used to obtain an estimate of the VEA of this series for any value of *n*.

### 3.2 Organic species

**FIGURE 6:**
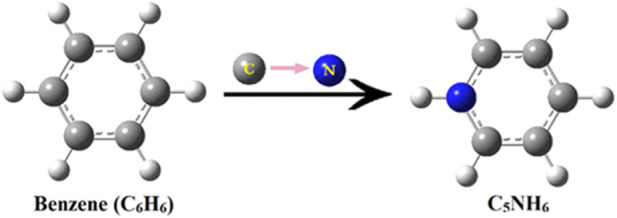
Turning benzene into organic superalkali.

Above non-metallic superalkalis were specifically based on the inorganic systems. However, non-metallic superalkalis are not only limited to inorganic species. There are several examples of carbon-based non-metallic superalkalis. We begin with the benzene, a classic example of organic aromatic species, following the Hückel’s rule of (4*n* + 2) *π*-electrons. The benzene (C_6_H_6_) possesses six *π*-electrons following this rule of aromaticity. Zhao et al. (Zhao et al., 2017) proposed that when a tetravalent carbon atom of C_6_H_6_ is replaced by a pentavalent nitrogen atom, the resulting system C_5_NH_6_ contains one extra π-electron beyond the Hückel’s rule of aromaticity as shown in Figure 6. This excess electron prompts C_5_NH_6_ to lose a *π*-electron to restore aromatic stability. The density functional calculations predicted the IE of C_5_NH_6_ to be 3.95 eV.

The work of Zhao et al. was further extended to pyrrole (C_4_H_5_N), which has the IE of nearly 8 eV, which led to the exploration of organic heterocyclic superalkalis. Reddy and Giri (Reddy and Giri, 2016) studied N-substituted pyrrole (C_3_H_5_N_2_), which was found to possess the IE of 4.3 eV, lower than that of Li (5.39 eV) and Na (5.14 eV). The authors also proposed that the substitution of methyl (CH_3_) in the place of H further reduces the IE such that it becomes as low as 3.0 eV for C_3_N_2_(CH_3_)_5_. They also suggested that other heterocycles (furan, thiophene, imidazole, pyrazole) can become superalkalis after H substitution with electron-donating groups, but not electron withdrawing groups. This idea was further extended in a subsequent study by Giri et al. (Giri et al., 2016). They validated their density functional results with those obtained with the MP2-methods. Zintl ions are multiply charged anions of group 13–15 elements. The authors employed heptaphosphide Zintl ion (P_7_^3−^) with ligands H, CHO, Me, CH_2_Me, CH(Me)_2_, C (Me)_3_, where Me represents methyl (CH_3_). The calculated IE of P_7_H_4_ (5.5 eV) was found to be close to Li. However, it was reduced to 4.79 eV (below Na) for P_7_ (Me)_4_. With the further substitution of Me in the place of H, the IE was obtained as low as 4.27 eV for P_7_C [(Me)_3_]_4_ (lower than K). On the contrary IE of P_7_(CHO)_4_ was found to be 6.61 eV, prohibiting it to be superalkali due to electron-withdrawing nature of CHO.

**FIGURE 7:**
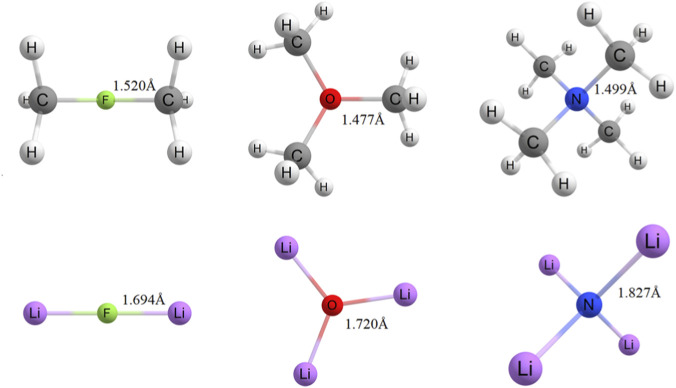
Equilibrium structures of X (CH_3_)_*k*+1_^+^ and XLi_*k*+1_^+^ superalkali cations from Ref. (Srivastava et al., 2022). The corresponding bond lengths are also indicated.

**FIGURE 8:**
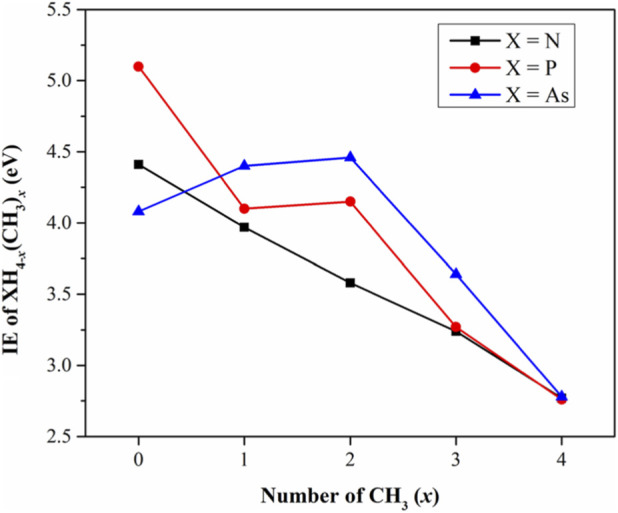
Variation of ionization energy of XH_4-*x*_ (CH_3_)_*x*_ as a function of *x* from Ref. (Srivastava and Srivastava, 2023).

The potential of CH_3_ in reducing the IE value can be attributed to its electron donating behaviour. Srivastava et al. (Srivastava et al., 2022) performed a systematic study on XLi_*k*+1_^+^ cations by replacing Li by CH_3_ groups and compared them with the parent species as shown in Figure 7. They benchmarked the MP2 method against available experimental data. For instance, the MP2-calculated VEAs of FLi_2_^+^ and OLi_3_^+^ cations 3.63 eV and 3.46 eV are in good agreement with the corresponding experimental values of 3.8 ± 0.2 eV (Veličković et al., 2007) and 3.54 ± 0.30 eV (Wu et al., 1979), respectively. From Figure 7, one can see that the structures of X (CH_3_)_*k*+1_^+^ are similar to those of corresponding XLi_*k*+1_^+^ cations. However, the order of bond-lengths is reversed. For instance, the bond length increases from FLi_2_→OLi_3_→NLi_4_ whereas it decreases from F(CH_3_)_2_→O(CH_3_)_3_→N(CH_3_)_4_. They noticed that the VEA values of CH_3_-substituted cations become 4.35 eV for F(CH_3_)_2_^+^, 2.87 eV for O(CH_3_)_3_^+^ and 2.76 eV for N(CH_3_)_4_^+^. These values are somewhat lower than those of corresponding Li species. The authors also shown that most of cations having O and N core with one or more CH_3_ ligands should behave as superalkalis due to their lower VEAs than that of Li or Na or K (if not, Cs). Later Srivastava and Srivastava (Srivastava and Srivastava, 2023) generalized this idea and studied XH_4-*x*_ (CH_3_)_*x*_ (*x* = 0–4) using X = N, P, and As atoms. Their calculated N–H bond length, 1.024 Å was in good agreement with the experimental data for N–H in the gaseous phase (N–H = 1.021 ± 0.002 Å) (Crofton and Oka, 1987). The calculated IE of these species are plotted in Figure 8. The IE of NH_3_CH_3_ (3.97 eV) is found to be comparable to that of Cs atom (3.89 eV). With an increase in CH_3_ groups, there is further decrease in the value of IE e.g., 3.58 eV for NH_2_(CH_3_)_2_ and 3.24 eV for NH(CH_3_)_3_, and 2.77 eV is the lowest IE of the structure N(CH_3_)_4_. Thus, there is monotonically decreasing trend in the IE of NH_4-*x*_ (CH_3_)_*x*_ with an increase in *x*. Similarly, the IE of PH_4-*x*_ (CH_3_)_*x*_ decreases from 5.10 to 2.76 eV. Likewise, AsH_4-*x*_ (CH_3_)_*x*_ follows the similar trend of IE values with the lowest value of 2.78 eV for As(CH_3_)_4_. This proves that the methylation of central core is an effective strategy in designing potential superalkali cations. Can it be generalized to alkylation? This was addressed by a density functional study of Srivastava (Srivastava, 2021) performed on 1-alkyl-3-methylimidazolium (*n*MIM).

**FIGURE 9:**
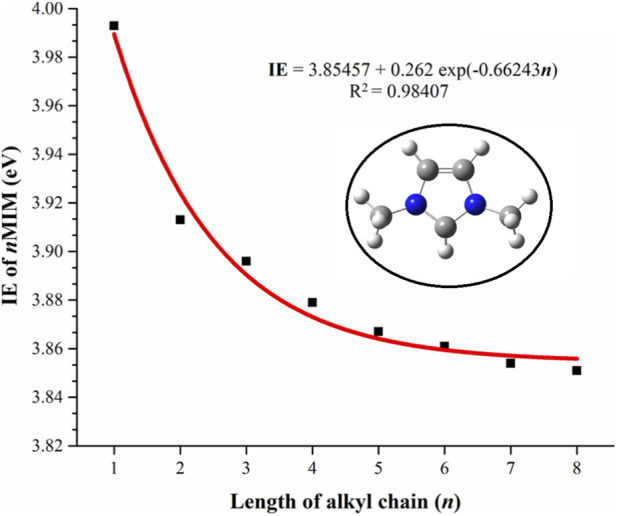
Variation of ionization energy of 1-alkyl-3-methylimidazolium (*n*MIM) as a function of *n* from Ref. (Srivastava, 2021).

Srivastava (Srivastava, 2021) used 1-methyl-3-methylimidazolium (1MIM) as shown in Figure 9. The IE of 1MIM is 3.99 eV. The variation of IE of *n*MIM is also displayed in Fig, 9, which shows gradual but consistent decrease in an increase in *n*. For instance, the IE decreases and reaches 3.85 eV for *n* = 8. There is an exponential statistical correlation between IE of *n*MIM and *n* with the correlation coefficient of 0.98407. The author also suggested further decrease by alkylation of both methyl groups of 1MIM. He calculated that the IE of 1-octyl-3-octylimidazolium (8OIM) to be 3.68 eV at the same level of theory. This suggested that the IE of imidazolium can be reduced effectively by simultaneous successive substitution of an alkyl groups in the ring.

## 4 Conclusion and future directions

**FIGURE 10:**
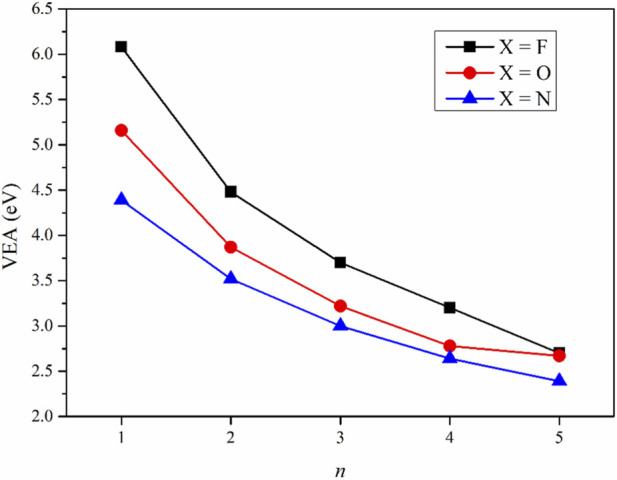
The vertical electron affinity (VEA) of X_*n*_H_*nk*+1_^+^series as a function of *n*.

We have demonstrated how the series of polynuclear superalkalis can be generated using non-metallic FH_2_^+^, OH_3_^+^, NH_4_^+^ cations and by substituting for H-atoms, successively. The resulting cations are stabilised by partially covalent and ionic X–H···X type H-bonds (X = F, O, N) of medium to weak strengths. These cationic series are energetically stable against loss of proton and loss of HF, H_2_O or NH_3_ as the case may be. The VEA of X_*n*_H_*nk*+1_^+^series (*k* is the valence of X) is plotted in Figure 10 for a visual comparison. One can see that the VEA X_*n*_H_*nk*+1_^+^series decreases monotonically with an increase in *n*, irrespective of X. However, the range of VEA values decreases as*k* increases. This is due to the fact that a larger number of H-atoms leads to more charge transfer to the central core.

Beyond inorganic species, it was shown that nitrogen substituted benzene yields C_5_NH_6_ with an IE of 3.95 eV, while pyrrole derivatives and methyl-substituted heterocycles further reduce IEs to values below those of alkali atoms. Zintl ions decorated with methyl ligands also display superalkali behavior, whereas electron-withdrawing groups suppress it. Srivastava and colleagues demonstrated that replacing Li with CH_3_ in cations lowers vertical electron affinities. Extending this principle, alkylation of imidazolium cations (*n*MIM series) shows an exponential correlation between chain length and IE reduction, with 1-octyl-3-octylimidazolium reaching 3.68 eV. Collectively, these studies establish methylation and alkylation as general strategies for designing organic superalkalis. Carbon-based C_*n*_H_4*n*+1_^+^ series has also been studied (Srivastava, 2020), which leads to similar results. This series is unique because of its organic nature. In principle, the series could be continued to obtain stronger superalkali cations with even lower VEA values, subject to the limitation of steric repulsions. Although we provided some ways to estimate the lower limit, an exact lowest value of VEA for any series requires a lot of effort.

These non-metallic series of superalkali cations, due to their “metallic” nature, might be capable of originating new chemistry. The stronger superalkali cations with extremely low VEA values will eventually have exceptional reducing capabilities and achieve notable theoretical milestones in the upcoming years. The exploitation of these cations in diverse applications, ranging from charge-transfer salts to supersalts, is yet to come. Some of these aspects are currently being explored in our lab. Being hydrogen-rich, these cations can be proven to be a virtue for chemical hydrogen storage as reported by us only recently (Srivastava, 2024).

Despite enormous progress in computational predictions, there is a lack of general benchmark for study of these systems. On the other hand, the experimental validation of superalkalis remains largely limited to simpler systems, primarily detected via mass spectrometry. A significant gap persists in the synthesis and structural characterisation of complex and/or newly designed superalkali species. Therefore, intensified efforts are needed to bridge the gap between theoretical design and experimental realization. Moreover, the stability of superalkali cations in solvent environments such as water, which has received limited attention, demands further investigation. Understanding solvation effects could be pivotal in expanding their applicability in practical systems. Although the fundamental understanding of superalkalis has advanced substantially, their translation into practical materials and technologies remains a fertile ground for future exploration.
